# Evolutionary drivers of protein shape

**DOI:** 10.1038/s41598-019-47337-8

**Published:** 2019-08-15

**Authors:** Gareth Shannon, Callum R. Marples, Rudesh D. Toofanny, Philip M. Williams

**Affiliations:** 1 NASA Ames Research Center, Moffett Field, CA 94043 USA; 2 Molecular Therapeutics and Formulation, School of Pharmacy, University of Nottingham, Nottingham, NG7 2RD United Kingdom; 3 Arzeda Corp., 3421 Thorndyke Ave W, Seattle, WA 98119 USA

**Keywords:** Molecular biophysics, Molecular evolution

## Abstract

Diffusional motion within the crowded environment of the cell is known to be crucial to cellular function as it drives the interactions of proteins. However, the relationships between protein diffusion, shape and interaction, and the evolutionary selection mechanisms that arise as a consequence, have not been investigated. Here, we study the dynamics of triaxial ellipsoids of equivalent steric volume to proteins at different aspect ratios and volume fractions using a combination of Brownian molecular dynamics and geometric packing. In general, proteins are found to have a shape, approximately Golden in aspect ratio, that give rise to the highest critical volume fraction resisting gelation, corresponding to the fastest long-time self-diffusion in the cell. The ellipsoidal shape also directs random collisions between proteins away from sites that would promote aggregation and loss of function to more rapidly evolving nonsticky regions on the surface, and further provides a greater tolerance to mutation.

## Introduction

Interactions between diffusing proteins are central to the function of the cell. The rate at which reactions of proteins occur in the cell is proportional to the product of their concentration and their rate of diffusion^1^. As protein concentration increases their translational diffusion ${D}_{t}^{0}$ slows due to interactions in the increasingly crowded environment^2^. For a system of hard spheres, which undergo a glass transition at a volume fraction *ϕ*_c_ ≈ 0.58^3^, the translational diffusion constant falls with volume fraction *ϕ* approximately as ${D}_{t}={D}_{t}^{0}{(1-\varphi /{\varphi }_{\text{c}})}^{2}$^4^. The product of diffusion constant and concentration is maximum when *ϕ* = 19%^1,2^, a value similar to that of proteins in the cell^5–7^.

The diffusion constant of a protein depends on its size and shape and on the shape of other macromolecules in its environment^8^. The translational diffusion constant of a spherical particle is proportional to its hydrodynamic radius, and the diffusion constant is smaller for an aspherical particle compared to the sphere of the same volume. Spherical proteins with their larger diffusion constants would, therefore, be expected to give rise to higher biochemical reaction rates than aspherical proteins of equivalent volume. Proteins are, however, generally not spherical^9^.

The glass transition point *ϕ*_c_ also depends on particle shape. Mode coupled theory predicts that *ϕ*_c_ is higher for spheroids (ellipsoids of revolution) than it is for spheres, suggesting spheroids prevent crystallization by raising the glass transition point^10^, and that *ϕ*_c_ of dumbbell-shaped particles increased with asymmetry up to a maximum when their length/diameter is around 1.4^11–13^. Simulations of the liquid-to-solid phase transition also suggest that *ϕ*_c_ is higher for spheroids that it is for spheres^14,15^. Raising the value of *ϕ*_c_ for protein would increase the diffusion constant and biochemical reaction rate. Studying the relationship between particle shape and *D*_*t*_ and *ϕ*_c_ and hence the possible relationships between protein shape, their concentration and their reactions within a cell is the subject of this work.

Here, we first study the aspect ratios of globular proteins by taking both calliper measurements and determining triaxial ellipsoids of equivalent steric volume and diffusive properties. Using a combination of molecular dynamics and ellipsoidal packing and unpacking simulations we show how the critical volume fraction and translational diffusion of these triaxial ellipsoids depends on their shape and concentration. Assuming the diffusion of proteins follows that predicted for these ellipsoidal models these simulations suggest that proteins have evolved an ellipsoidal shape that limits aggregation and gelation in situations of high concentration, and to maximise diffusion-limited processes within the cell. We further analyse the collisions between diffusing ellipsoids and suggest that proteins have evolved binding sites on their surface at regions that limit the likelihood of aggregation during collisions.

## Materials and Methods

47 677 structures from the Protein Data Bank clustered at 95% sequence identify using CD-HIT were downloaded from RCSB.org on 01/23/2018. The proteins in this set were taken as the single-chain, and multimerisation ignored. The 3D Complex^16^ (single chain proteins and protein dimers), PDB Select (asymmetric units)^17^, Dynameomics^18^, and species^19^ subsets were also studied.

### Calliper measurements

To determine the extent of asphericity of proteins Feret diameters were measured by determining the largest and smallest differences between the maximum and minimum atomic coordinate values in the *x*, *y*, and *z* planes for all orientations of a protein around the *x* and *y* planes between 0 and *π*/2 in *π*/180 increments. The maximum error in this estimation is 1 − cos(*π*/180) = 0.02%.

### Ellipsoid of equivalent steric volume

To calculate the principle radii of the ellipsoid of the equivalent steric volume to a protein we calculated the moment of inertia of a body representing the atoms^20^

1 $$I=\frac{4\pi }{3}\mathop{\sum }\limits_{i\mathrm{=1}}^{N}\,{r}_{i}^{3}[\begin{array}{ccc}{y}_{i}^{2}+{z}_{i}^{2}+\frac{2}{5}{r}_{i}^{2} & -{x}_{i}{y}_{i} & -{x}_{i}{z}_{i}\\ -{x}_{i}{y}_{i} & {x}_{i}^{2}+{z}_{i}^{2}+\frac{2}{5}{r}_{i}^{2} & -{y}_{i}{z}_{i}\\ -{x}_{i}{z}_{i} & -{y}_{i}{z}_{i} & {x}_{i}^{2}+{y}_{i}^{2}+\frac{2}{5}{r}_{i}^{2}\end{array}]$$

where *x*, *y*, and *z* are the positions of the *N* atoms in the protein, and *r* is their vdW-radii (C 1.7 Å; N 1.55 Å; O 1.52 Å; S 1.8 Å; H 1.2 Å; other 1.7 Å). This tensor is diagonalized to give the three eigenvectors of the principle axes and their eigenvalues *λ*_1_ ≥ *λ*_2_ ≥ *λ*_3_. The lengths *a*, *b*, and *c* of the semi-axes of the ellipsoid of equivalent steric volume are then found as

2 $$a=\sqrt{\frac{5}{2M}({\lambda }_{1}+{\lambda }_{2}-{\lambda }_{3})},$$

3 $$b=\sqrt{\frac{5}{2M}({\lambda }_{1}+{\lambda }_{3}-{\lambda }_{2})},$$

4 $$c=\sqrt{\frac{5}{2M}({\lambda }_{2}+{\lambda }_{3}-{\lambda }_{1})},$$

where $M={\sum }_{i=1}^{N}\,\frac{4\pi }{3}{r}_{i}^{3}$. Here, we express the ratios of these axes lengths by the parameters *α* = *a*/*c*, the aspect ratio of the ellipsoid, and *α*^*β*^ = *b*/*c*, which describes the shape from prolate (*β* = 0) to oblate (*β* = 1) spheroid. Code for this was written in C using diagonalization routines of Kopp^21^ (http://arXiv:physics/0610206).

### Brownian and Langevin dynamics

HOOMD-blue (v2.1.1-92)^22,23^ was used to simulate the diffusive motion of soft spheroids at volume fractions between 0.001% and 55%. An initial starting configuration was generated by packing 300 randomly oriented spheroids to a volume fraction of 30% using PackLSD (see below). The position and orientation of the packed spheroids were then used to generate 8100 Gay-Berne particles by replicating in a 3 × 3 × 3 array. The Gay-Berne anisotropic pair potential used in HOOMD-blue is^24^

$$\begin{array}{rcl}{V}_{{\rm{GB}}}(\overrightarrow{r},{\overrightarrow{e}}_{i},{\overrightarrow{e}}_{j}) & = & \{\begin{array}{ll}4\varepsilon [{\zeta }^{-12}-{\zeta }^{-6}] & \zeta  < {\zeta }_{{\rm{cut}}}\\ 0 & \,{\rm{overwise}}\,\end{array}\\ \zeta  & = & (\frac{r-\sigma +{\sigma }_{{\rm{\min }}}}{{\sigma }_{{\rm{\min }}}})\\ {\sigma }^{-2} & = & \frac{1}{2}\hat{\overrightarrow{r}}\cdot {\overrightarrow{H}}^{-1}\cdot \hat{\overrightarrow{r}}\\ \overrightarrow{H} & = & 2{\ell }_{\perp }^{2}\overrightarrow{1}+({\ell }_{||}^{2}-{\ell }_{\perp }^{2})({\overrightarrow{e}}_{i}\otimes {\overrightarrow{e}}_{i}+{\overrightarrow{e}}_{j}\otimes {\overrightarrow{e}}_{j})\\ {\sigma }_{{\rm{\min }}} & = & {\rm{\min }}({\ell }_{\perp },{\ell }_{||})\end{array}$$

with ${\ell }_{||}$ and ${\ell }_{\perp }$ set such that *V*(2*a*) = *V*(2*c*) = 1

Dynamics of unit-volume spheroids was performed for 10 000 steps with *k*_B_*T* = 1, translational and rotational friction factors *γ* = 1, timestep *δt* = 0.0001 whilst the size of the periodic box was changed to reach the specified volume fraction, the system equilibrated for a further 10 000 steps and then particle diffusion monitored over 250 000 steps (*t* = 25). Translational diffusion was determined from the mean-squared displacement *d* of the particles *D*_t_ = <*d*^2^>/6*t*. Eq. (14) was fitted to the simulated diffusion data to determine *ϕ*_c_ using OriginPro (OriginLab Corporation, Northampton, MA 01060).

In-house code was used to study the location of collisions between ellipsoids in Brownian motion described by Eq. (5) and Eq. (6). Here, a point *x*, *y*, *z* on the surface of an ellipsoid in its body frame of reference (i.e. axis *a* aligns with *x*, *b* with *y* and *c* with *z*) is defined by two angles *θ* and *φ*, where *x* = *t* cos(*θ*), *y* = *t* sin *θ* cos(*φ*), *z* = *t* sin(*θ*) sin (*φ*) with *t* given by

$$t=\frac{abc}{\sqrt{{b}^{2}{c}^{2}\,{\cos }^{{\rm{2}}}\,(\theta )+{a}^{2}{c}^{2}\,{\sin }^{2}(\theta )\,{\cos }^{{\rm{2}}}(\varphi )+{a}^{2}{b}^{2}\,{\sin }^{2}(\theta )\,{\sin }^{2}(\varphi )}}\mathrm{.}$$

### Packing and unpacking

Maximally random jammed packings of ellipsoids were generated using the neighbour list collision-driven molecular dynamics algorithm PackLSD of Donev^25^. Unpackings were performed by running PackLSD on an ordered array of ellipsoids, as described in ref.^26^, to decompress to a final volume fraction of 35%.

### Estimation of the critical volume fraction *ϕ*_c_ for ellipsoids

To estimate the critical volume fraction *ϕ*_c_ we studied the liquid-to-solid phase transition of triaxial ellipsoids. For a system of hard spheres the phase diagram of pressure as a function of volume fraction exhibits a freeze point *ϕ*_F_ ≈ 0.494, below which the suspension is a liquid^27^. Between *ϕ*_F_ and *ϕ*_M_ ≈ 0.545, the melt point, for a system in equilibrium solid and liquid coexist and above which the system is a crystal. Forcing a system above *ϕ*_F_ quickly enough to preclude equilibration sees the system enter a supercooled state of liquid until the glass transition *ϕ*_G_ ≈ 0.58 is reached, and between this and the maximally random jammed stated *ϕ*_MRJ_ ≈ 0.64 the system behaves as a glass^14^. There is some debate as which value is best for the glass transition: the value *ϕ*_G_ ≈ 0.58 or the maximally jammed state value *ϕ*_MRJ_ ≈ 0.64. Most experiments point to *ϕ*_G_ ≈ 0.58, for example eye-lens spherical *α*-crystallin multimers (*ϕ*_G_ = 0.579 ± 0.004)^28,29^, although some suggest the value of 0.64 is the true value^30,31^. Santamaria-Holek and Mendoza used *ϕ*_c_ = *ϕ*_MRJ_ when predicting the relative viscosity of ellipsoids^32^. Here, we assumed *ϕ*_c_ = *ϕ*_G_, regardless of its value.

To estimate *ϕ*_c_ for triaxial ellipsoids we followed the method of Donev where a dense crystal arrangement of packed ellipsoids is unpacked and their order parameter and the pressure of the system monitored^26^. As the system was unpacked, the point at which order is lost precipitously was taken as the freeze point, *ϕ*_F_, which also corresponds to an increase in pressure^15,25^. We took the melt point, *ϕ*_M_, as the volume fraction of the unpacked ellipsoids that exerted the same pressure as this raised value of the freeze point. The value of *ϕ*_MRJ_ was determined by packing the ellipsoids from the random (liquid) state obtained at the end of the unpacking. We then studied how ellipsoid diffusion varied when taking the values found for *ϕ*_F_, *ϕ*_M_ and *ϕ*_MRJ_ as *ϕ*_c_.

### Mapping

Maps of the location of contacts between ellipsoids and of residue type were generated by representing each *θφ* contact point as a Gaussian spot. To calculate the Gaussian, geodesic distances on the surface of the ellipsoid between two integer *θφ* points were estimated by considering the map as an image of 180 × 360 pixels, where each represents the integer degree value of the angles. All pixel values were initially set to zero. Starting at the pixel corresponding to the first point, the cartesian distance to the centres of its nearest unvisited neighbouring pixels was calculated at the closest found. The closest pixel was then given the value of the sum of its current value and this distance. Next, the pixel with the lowest value and with unvisited neighbours was selected. This process was iterated until the value of the pixel corresponding to the second point was set, which was the approximation of the geodesic distance from the first.

### Positional evolutionary rates

The rates of residue evolution were calculated for the 382 orthologous sequences taken from the genomes of *Saccharomyces cerevisiae* and nine closely related species: *Saccharomyces paradoxus*, *Saccharomyces mikatae*, *Saccharomyces bayanus*, *Candida glabrata*, *Saccharomyces castellii*, *Saccharomyces kluyveri*, *Kluyveromyces lactis*, *Kluyveromyces waltii*, and *Ashbya gossypii* using Rate4Site^33^ as described in ref.^34^. As in that work, evolutionary rates are normalised to the average of all positions in all proteins in the set, and presented as ${\mathrm{log}}_{2}[{\rm{normalisedrate}}]$.

### Equations of diffusion

#### Rotational and diffusional constants of triaxial ellipsoids

Many methods have been derived to predict the translational and rotational diffusion of proteins^35–39^. Here, using the semi-axis lengths of the ellipsoid of equivalent steric volume we calculated the translational ${D}_{{\rm{t}}a,b,c}^{0}$ and rotational ${D}_{{\rm{r}}a,b,c}^{0}$ diffusion coefficients along and around each semi-axis as (in the example of *a*)^40^

5 $${D}_{{\rm{t}}a}^{0}=\frac{{k}_{B}T}{6\pi \eta }(\frac{3{R}_{F}({a^{\prime} }^{2},{b^{\prime} }^{2},{c^{\prime} }^{2})+{R}_{D}({b^{\prime} }^{2},{c^{\prime} }^{2},{a^{\prime} }^{2}){a^{\prime} }^{2}}{4}),$$

6 $${D}_{{\rm{r}}a}^{0}=\frac{{k}_{B}T}{8\pi \eta }(\frac{{R}_{D}({c^{\prime} }^{2},{a^{\prime} }^{2},{b^{\prime} }^{2}){b^{\prime} }^{2}+{R}_{D}({a^{\prime} }^{2},{b^{\prime} }^{2},{c^{\prime} }^{2}){c^{\prime} }^{2}}{{b^{\prime} }^{2}+{c^{\prime} }^{2}}),$$

respectively, for a protein where *a*′, *b*′ and *c*′ are the semi-axis lengths of the equivalent ellipsoid increased by an amount *δH* to reflect the width of a stationary hydration layer surrounding the protein, and *R*_*F*_ and *R*_*D*_ are the Carlson symmetric elliptic integrals of the first *R*_*F*_(*x*, *y*, *z*), and second *R*_*D*_(*x*, *y*, *z*) kind

7 $${R}_{F}(x,y,z)=\frac{1}{2}{\int }_{0}^{\infty }\,\frac{d\lambda }{\sqrt{(x+\lambda )(y+\lambda )(z+\lambda )}},$$

8 $${R}_{D}(x,y,z)=\frac{3}{2}{\int }_{0}^{\infty }\,\frac{d\lambda }{(z+\lambda )\sqrt{(x+\lambda )(y+\lambda )(z+\lambda )}}.$$

The algorithms to solve these were taken from *Numerical Recipes*^41^. The long-time translational and rotational diffusion constants are the arithmetic mean of the values for each axis

9 $${D}_{{\rm{t}}}^{0}=({D}_{{\rm{t}}a}^{0}+{D}_{{\rm{t}}b}^{0}+{D}_{{\rm{t}}c}^{0})/3,$$

10 $${D}_{{\rm{r}}}^{0}=({D}_{{\rm{r}}a}^{0}+{D}_{{\rm{r}}b}^{0}+{D}_{{\rm{r}}c}^{0})/3.$$

In terms of *α*′ = (*a* + *δH*)/(*c* + *δH*), *α*′^*β*′^ = (*b* + *δH*)/(*c* + *δH*) and $r^{\prime} ={(a^{\prime} b^{\prime} c^{\prime} )}^{\frac{1}{3}}$

11 $${D}_{{\rm{t}}}^{0}=\frac{{k}_{B}T}{6\pi \eta r^{\prime} }{R}_{F}({({\alpha ^{\prime} }^{2-\beta ^{\prime} })}^{\frac{2}{3}},{({\alpha ^{\prime} }^{2\beta ^{\prime} -1})}^{\frac{2}{3}},{({\alpha ^{\prime} }^{\beta ^{\prime} +1})}^{-\frac{2}{3}}).$$

#### Diffusion at finite concentration

Interactions between diffusing bodies leads to diffusive motion that is dependent on timescale and concentration^42^. There are several descriptions of the correlation between intrinsic viscosity or long-time diffusion of spheres and volume fraction, all sharing a critical volume fraction at the divergent point^43–45^. Tokuyama *et al*. described the short-time self-diffusion of spheres by^4^

12 $${D}_{S}^{S}(\varphi )=\frac{{D}_{t}^{0}}{1+L(\varphi )},$$

where *ϕ* is the volume fraction of the particles in the suspension and *L*(*ϕ*) defined as

13 $$L(\varphi )=\frac{2{B}^{2}}{1-B}-\frac{C}{1+2C}-\frac{BC(2+C)}{(1+C)(1-B+C)},$$

with *B* = (9*ϕ*/8)^1/2^ and *C* = 11*ϕ*/16. At longer times, a cage-effect of proteins surrounding others decreases diffusion further with proteins needing to transit from cage to cage. The long-time self-diffusion coefficient of a soft particle is described well by the expression

14 $${D}_{S}^{L}(\varphi ,{\varphi }_{{\rm{c}}})=\frac{{D}_{S}^{S}(\varphi )}{1+\kappa \frac{{D}_{S}^{S}(\varphi )}{{D}_{{\rm{t}}}^{0}}(\frac{\varphi }{{\varphi }_{{\rm{c}}}}){(1-\frac{\varphi }{{\varphi }_{{\rm{c}}}})}^{-2}},$$

where *ϕ*_c_ is a singular point of the cessation of long-time self-diffusion; the critical volume fraction. Eqs (12) and (14) with a value *κ* = 2 have been shown to describe the volume fraction dependence of translational diffusion for a number of proteins, where the function has been fitted to the experimental data to determine the value of *ϕ*_c_^44,46–48^.

## Results and Discussion

### Proteins are naturally aspherical and have an aspect ratio around 1.6

**Figure 1:**
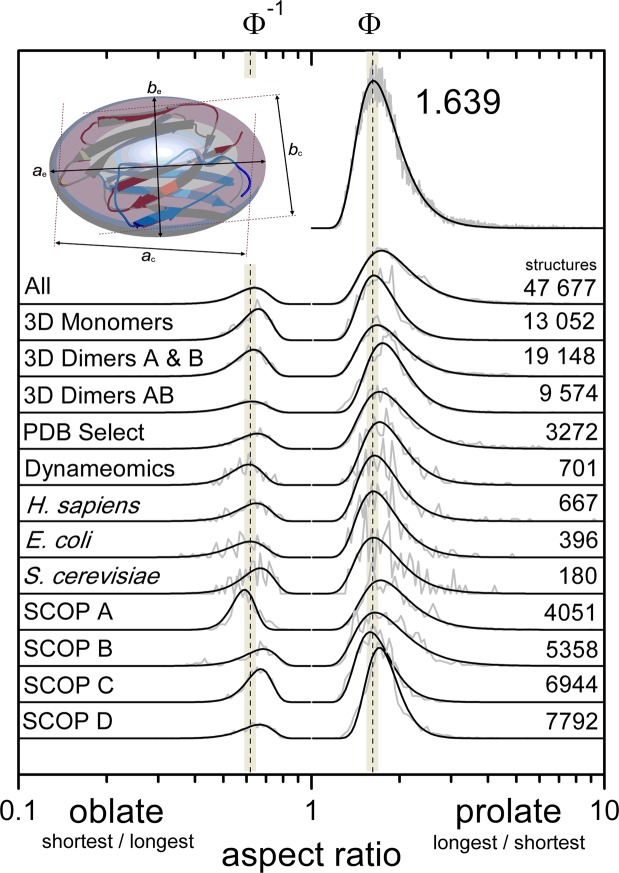
The aspect ratio of proteins taken from calliper measurements *a*_*c*_ and *b*_*c*_ distribute around a modal value of 1.6. The ratio of the longest to shortest calliper measurements for 47 677 structures (single monomer chains) taken from the PDB database form a distribution around a modal value of 1.639. By determining the ellipsoid of equivalent steric volume the proteins can be separated into prolate and oblate-shaped structures. The distributions of aspect ratio of proteins in subsets of the PDB database (see text) are shown for oblate as *b*_*c*_/*a*_*c*_ and prolate as *a*_*c*_/*b*_*c*_ proteins.

The aspect ratio of proteins, calculated as the ratio of the largest-to-smallest calliper diameters, is broadly distributed around 1.6 and ranges from 1.2 to 18.6 (Fig. 1). The distribution of aspect ratio of the single chains of 47 677 PDB structures clustered at 95% sequence similarity is represented well by a log-lognormal with a modal value of 1.639 (±0.001). We divided the aspect ratio distributions into those of proteins that are generally prolate and of those generally oblate by determining the moment of inertia of the protein represented by its atoms as unit density van-der Waals radius spheres and determining the semi-axis lengths *a* ≥ *b* ≥ *c* of the ellipsoid with the same inertia; the so-called ellipsoid of equivalent steric volume^20^. We found the aspect ratio of the calliper measurements as longest-to-shortest of prolate and shortest-to-longest of oblate are distributed around 1.6 and 0.7 ($\sim 1/1.6$), respectively (Fig. 1 All). The modal aspect ratios are similar to that of the Golden ratio ${\rm{\Phi }}=\mathrm{(1}+\sqrt{5})/2=1.618\ldots$, and its reciprocal Φ^−1^. The distributions of aspect ratios of the 13 052 monomers (3D Monomers), 19 148 dimer components (3D Dimers A & B) and their 9574 dimeric complexes (3D Dimers AB) of the structures in 3D Complex database^16^, 3272 single chain asymmetric units in the PDBselect database^17^, 701 proteins in the Dynameomics database^18^, and 1243 proteins from different species (667 *H. sapiens*; 396 *E. coli*; 180 *S. cerevisiae*)^19^ are similarly shaped (Fig. 1). The modes of these distributions are given in Table 1.

**Table 1:** Calliper aspect ratios and shape parameters measured for various subsets of proteins in the PDB.

Dataset	Number	Calliper		Equivalent	
		oblate	prolate	ellispoid	
		short/long	long/short	*α*	*β*
All PDB (single chains)	47 677	0.649	1.676	**1.65**	**0.34**
D Complex^16^ (dimers A & B)	19 148	0.635	1.676	1.54, **1.78**	0.44, **0.44**
D Complex^16^ (dimers AB)	9 574	0.627	1.749	**1.93**	**0.17**
D Complex^16^ (monomers)	13 052	0.659	1.612	1.35, **1.65**	0.45, **0.30**
PDBselect^17^	3272	0.664	1.657		
Dynameomics^18^	701	0.620	1.665		
*H. sapiens* ^19^	667	0.658	1.602		
*E. coli* ^19^	396	0.630	1.588		
*S. cerevisiae* ^19^	180	0.678	1.583		

**Figure 2:**
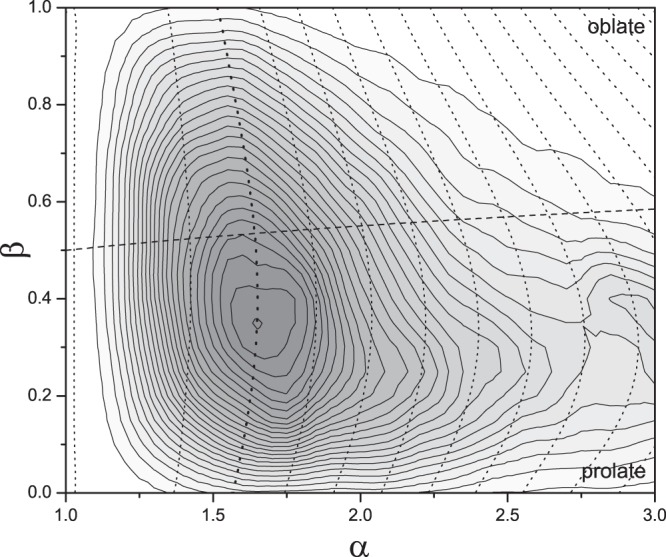
The distribution of the aspect ratios for the ellipsoids of equivalent steric volume of the 47 677 structures taken from the PDB database (single chains clustered at 95% sequence similarity). For each ellipsoid with semi-axis lengths *a* ≥ *b* ≥ *c*, *α* = *a*/*c* and *α*^*β*^ = *b*/*c*. The Gaussian-smoothed distribution has a maximum at *α* = 1.65, *β* = 0.34. The dashed line left-to-right for *α* > 1 shows the boundary between generally prolate (*β* = 0) and generally oblate (*β* = 1) structures. 75% of the structures are generally prolate. The dotted isoperimetric contour lines between values of *β* represent ellipsoids of equal volume and surface area.

This separation of the distribution shows that approximately 25% of proteins are oblate, a value similar to that found by Dima and Thirumalai who studied the proteins in the PDBselect^17^ subset of the Protein Data Bank^9^. As an unfolded chain is generally prolate we may question why some proteins fold to oblate structures as such would need to undergo significant changes in volume and/or surface area on folding^49–51^. This misconception arises from the approximation to purely oblate and prolate spheroids of revolution that suggests the change between the two requires a transition through a spherical state with corresponding differences in surface area or volume. The distribution of protein shape is better represented in two dimensions of *αβ*, where *α* = *a*/*c* and *α*^*β*^ = *b*/*c*. Figure 2 shows this 2D distribution for the 47 677 protein chains. The dashed line *β* = (ln(*α* + 1) − ln(2))/ln(*α*) for *α* > 1 shows the boundary between prolate (*β* = 0; lower) and oblate (*β* = 1; upper) proteins. We also plot (dotted) contours of the isoperimetric quotient (36*πV*^2^/*A*^3^) that represent ellipsoids of equivalent surface area and volume^52^. The *αβ* aspect ratios of proteins are broadly distributed around a modal value of *α* = 1.65 and *β* = 0.34. These values of aspect ratio correspond to the aspericity parameter Δ ≈ 0.1 reported in ref.^9^.

The surface area *A* of an ellipsoid defined by *α*, *β* and *c* with volume $V=\frac{4}{3}\pi {\alpha }^{(1+\beta )}{c}^{3}$ is approximately

15 $$A\approx {(\frac{6\sqrt{\pi }V}{{\alpha }^{1+\beta }})}^{\frac{2}{3}}{(\frac{{\alpha }^{\mathrm{(1}+\beta )p}+{\alpha }^{p}+{\alpha }^{\beta p}}{3})}^{\frac{1}{p}},$$

with p = 1.6075, which for all values of *β* increases monotonically with *α* from the smallest (sphere) value at *α* = 1. For an ellipsoid of fixed volume and surface area there is a unique value of *α* for each value of *β* between 0 and 1. Notwithstanding constraints due to necessary rearrangements of the polypeptide chain a molten globule could morph from prolate to oblate maintaining constant volume and surface area (i.e. along an isoperimetric contour). This suggests, therefore, that the shape of the folded protein may not necessarily reflect the general shape of its denatured state. Interestingly, *A* has a minimum in *β* between 0 and 1 for values of *α* > 1, so the transition between prolate and oblate spheroids at constant volume and surface area requires a seemingly paradoxical increase in aspect ratio *α*. A value of *α* > 1, where the surface area of the protein is greater than the sphere of equivalent volume, affords a greater tolerance to mutation than a spherical protein would, as any mutations which cause a change in volume can be accommodated without a change in surface area, and vice versa^53^.

**Figure 3:**
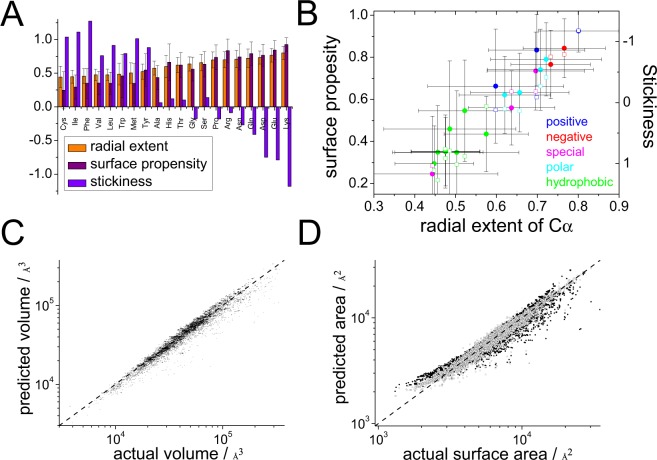
(**A**,**B**) The average fractional radial distance of the C_*α*_ of each type of residue from the core to the surface of a protein (A orange) is independent of protein shape and is ordered similarly to the frequency at which they are found at the surface (A magenta; B closed symbols), and opposite to their stickiness (A violet; B open symbols)^19^. (**C**) The volume of the ellipsoid of equivalent steric volume can be accurately predicted from the number of residues. (**D**) The number of residues at the surface of the protein can be predicted from the sum of each residue content and their surface propensities (partial least squares with one component, closed symbols; Bayesian Regularized Neutral Network with 20 neurons, open symbols).

The volume of the ellipsoidal approximation of protein was found to scale with the number of residues *N* as *V* ≈ 203*N* Å^3^ (Fig. 3C), equal to an equivalent radius of 3.1 Å-per-residue at a packing density of 0.64, and the surface area of this ellipsoid of equivalent steric volume approximates to *A* ≈ 47*N*_*s*_ Å^2^ (equivalent radius of 3.7 Å-per-residue at the maximum disc packing density of 0.9), where *N*_*s*_ is the number of residues at the surface. These findings are in agreement with those of others^9,54^. As expected, the extent to which amino acids are buried was found to be correlated with their hydrophobicity and, in general, anticorrelated with their ‘stickiness’ as defined in ref.^19^ (Fig. 3A,B).

**Figure 4:**
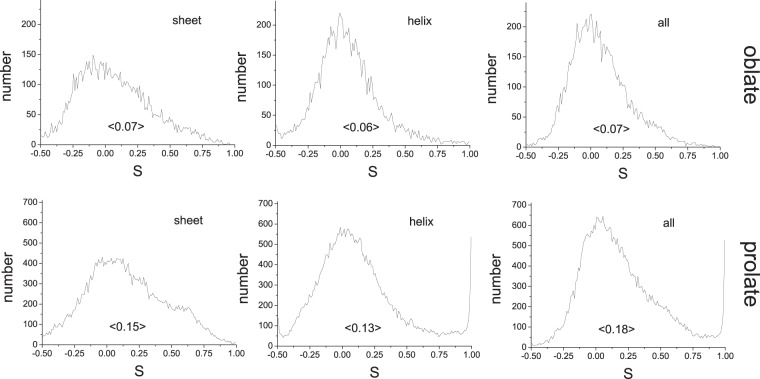
The order parameter $S= < \,1.5\,{\cos }^{2}\theta -0.5 >$ where *θ* is the angle between the vector defining the start (C_*α*_) and end of each secondary structural unit (left sheet; middle helix; right both) in a protein and the *a* axis of its ellipsoid of equivalent steric volume was calculated (top oblate; bottom prolate). Average values are shown in 〈brackets〉.

The average fractional radial extent of the alpha carbon of each residue toward the surface of the equivalent ellipsoid afforded an estimation of the likelihood that an amino acid of a type would be at the surface. By summing the product of these values by the number of each residue type in the protein we could estimate *N*_*s*_, and hence surface area (Fig. 3D). This estimation could be improved by using the actual frequencies of each residue type being at the surface. It is possible, therefore, to estimate both volume and surface area of a protein’s equivalent ellipsoid, and therefore, from Eq. (15) its aspect ratio from knowledge of the amino acid content alone. Since the volume of a protein is related to the number of amino acids and its surface area related to the amino acid composition, Eq. (15) reveals that a protein aspect ratio *α* is dependent on the fractional content of surface-exposed residues. This indicates that for a chain of a given length there are far more different amino acid compositions that give rise to ellipsoids than give rise to spheres, and therefore suggest that proteins are naturally ellipsoidal irrespective of secondary structure. To support this we calculated the order parameter $S= < \,1.5{\cos }^{2}\theta -0.5 >$ where *θ* is the angle between each secondary structural unit (helix or sheet) and the *a* axis of the protein (all, and separated into prolate and oblate structures) and found no correlation in alignment between secondary structure and the overall ellipsoidal shape of the protein (${\bar{S}}_{{\rm{all}}}=\mathrm{0.159,}\,{\bar{S}}_{{\rm{prolate}}}=0.183,{\bar{S}}_{{\rm{oblate}}}=0.072$) (Fig. 4).

**Figure 5:**
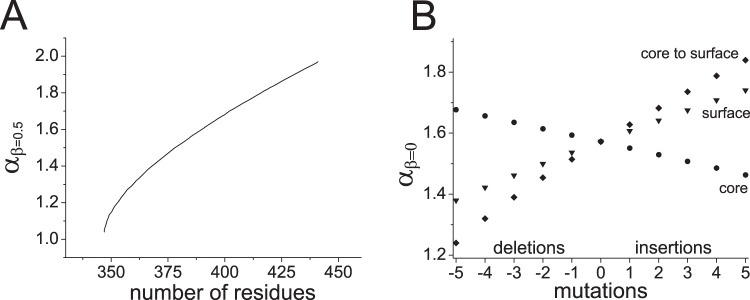
(**A**) Predicted aspect ratio *α*_*β*=0.5_ for proteins where half of their residues are on the surface. (**B**) A 400 residue ellipsoidal protein can accommodate deletions(−)/insertions(+) of residues at the surface (triangles), deletions(−)/insertions(+) in the core (circles), and rearrangements from surface-to-core(−)/core-to-surface+ (diamonds) by changing aspect ratio.

By considering the volume of the protein is related to its chain length and its surface area related to its composition we suggest that the general ellipsoidal shape of a protein (and its molten globule) is inherent. The fact that proteins can fold successfully without the assistance of chaperones^55^, and can successfully refold following denaturation and hence not co-translationally proximal to the ribosomal vestibule^56,57^, suggests that their shape is not defined by the physical environment in which they fold. We estimated the extent of this ellipticity of proteins by considering the random sequence polypeptide chain as a binary sequence of either surface (polar) or buried (hydrophobic) residues, similar to a HP polymer model, which are also seen to collapse into ellipsoidal shapes^58^. The binomial theorem shows that the largest number of combinations of H and P is when they are in equal amounts. The dependence of aspect ratio *α* on chain length of this simple model when *β* = 0.5 is shown in Fig. 5A. For a chain of 400 residues, equal to the average length in the human proteome, the most frequent composition has 200 surface exposed residues giving rise to ellipsoid aspect ratios for varying *β* of *α*_*β*=0_ = 1.57 to *α*_*β*=0.4_ = 1.66 to *α*_*β*=1_ = 1.52 (coincident with the contour line through the maximum of the distribution in Fig. 2). The ability of this model protein to accommodate mutations causing changes in volume and/or surface area is shown in Fig. 5B.

Taking *N*_*s*_/*N* to be 0.55, the value most found in the proteins and equal to the eleven out of twenty residues that have a surface preference value exceeding 0.5 (Fig. 3A), we find the length of protein where the predicted surface area is at least equal that of the sphere of equivalent volume is 36*π*(203)^2^/0.55^3^47^3^ = 270 (the value of *N* when in Eq. 1 *A* = 47(0.55*N*), *V* = 203*N*, and *α* = 1) residues. Proteins maintaining a 0.55/0.45 surface/core ratio of residues are predicted, therefore, to be at least approximately 270 residues in length. Larger proteins can be formed maintaining this surface/core ratio by becoming elliptical, but smaller proteins only formed through an increased prevalence of surface-preferred residues. Eukarya have median protein lengths longer at 361 residues, bacteria at 267 residues, and archaea are shorter at 247 residues^59^.

### The ellipsoids of equivalent steric volume allow the accurate prediction of protein diffusion

**Figure 6:**
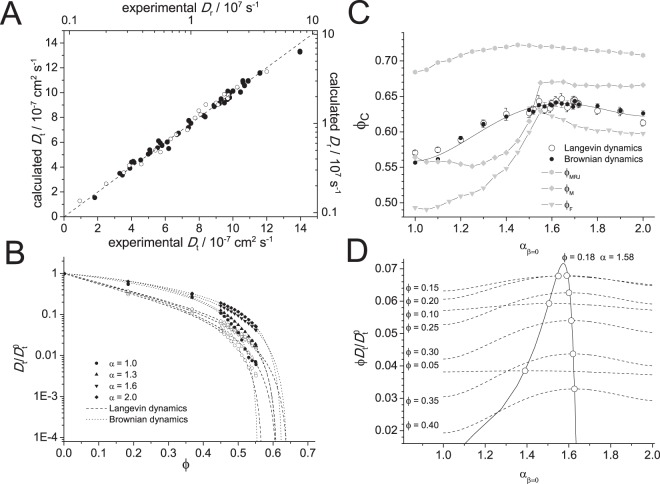
Protein diffusion is dependent on its ellipsoidal shape. (**A**) The translational diffusion constant ${D}_{{\rm{t}}}^{0}$ (solid) and rotational diffusion constant ${D}_{{\rm{r}}}^{0}$ (open) of 42 proteins is accurately predicted from their ellipsoids of equivalent steric volume dilated by 2.3Å. (**B**) Langevin and Brownian dynamics simulations of spheroids of varying aspect ratio reveal the reduction of translation diffusion with increasing volume fraction to the critical value *ϕ*_c_. (**C**) The value of *ϕ*_*c*_ taken from fits to the Langevin (open circles) and Brownian (closed circles) dynamics reveal the dependence on aspect ratio which rises from 0.56 for spheres to 0.64 for prolate spheroids of aspect ratio 1.6, which then falls with increasing asphericity. The calculated freeze *ϕ*_F_ (triangles), melt *ϕ*_M_ (diamonds), and maximum jammed *ϕ*_MRJ_ (hexagons) volume fractions for prolate spheroids (*β* = 0) are also shown. (**D**) The product of the volume fraction and the predicted diffusion constant for varying aspect ratios using Eq. (14) with *ϕ*_c_ values taken from a polynomial fit to the Langevin and Brownian dynamics values shown in (**C**) shows a maximum for spheroids with aspect ratio of 1.58 at a volume fraction of 0.18.

Calculated values of the translational and rotational diffusion constants of the equivalent steric triaxial ellipsoid of proteins, using Eqs (5) and (6), respectively, matched the experimentally determined values after increasing the semi-axis lengths of the ellipsoid by 2.32 Å, for PDB structures without hydrogens, or 2.30 Å for those with (translation rRMSE = 4.7%, rotation rRMSE = 9.1%) (Fig. 6A)^36,39^. Thus, the ellipsoid of equivalent steric volume with a hydration layer of 2.3 Å, equivalent to a hydration level of 0.38 g/g (volume of equivalent ellipsoid with boundary layer ≈ 2.39 *M*_p_, density of bound water 1.104 g/mL), facilitates the prediction of the translational and rotational diffusive properties of globular proteins, indicating the effective hydrodynamic radii of a protein for each of its semi-axes is equivalent to this ellipsoid of equivalent steric volume. The hydration value of 0.38 g/g is commensurate with a monolayer coverage of water. Taking the example of lysozyme with equivalent ellipsoid semi-axis lengths of *a* = 23.4 Å, *b* = 15.3 Å and *c* = 13.9 Å (*α* = 1.69, *β* = 0.19) we calculate a hydration shell volume of at most 9874 Å^3^ containing up to 365 water molecules. Microparticle dehydration studies estimate that lysozyme is hydrated by a stationary layer of ≈380 water molecules^60^.

By noting that the volume of the equivalent hydrated ellipsoid $\frac{4\pi }{3}{r^{\prime} }^{3}\approx 2.39\,{M}_{{\rm{p}}}$, and that *α* = 1.65 and *β* = 0.34, we can approximate the translational and rotational diffusion constants from protein length *N* or weight *M*_p_ as (20 °C, *ρ* = 1.0016 mPa s)

16 $${D}_{{\rm{t}}}^{0} \sim \frac{52}{\sqrt[3]{N}}\approx \frac{252}{\sqrt[3]{{M}_{{\rm{p}}}}}[\times {10}^{-7}\,{{\rm{cm}}}^{2}\,{{\rm{s}}}^{-1}],$$

17 $${D}_{{\rm{r}}}^{0} \sim \frac{9000}{{M}_{{\rm{p}}}^{0.9}}\approx \frac{20\,000}{{M}_{{\rm{p}}}}[\times {10}^{7}\,{{\rm{s}}}^{-1}].$$

The numerator in this approximation of the translational diffusion coefficient lies between the value of 244 given by Young-Carroad-Bell^35^ and 285 given by Polson^61^. It was noted by Hem and Neimeyer^38^ that the equation derived by Tyn and Gusek^36^ for the approximation of diffusion based on a protein’s radius of gyration implies a spheroidal geometry with an aspect ratio of 1.4 if prolate and 0.66 (1/1.5) if oblate.

### Diffusion within the crowded cell is greater for ellipsoidal proteins

The Brownian and Langevin dynamics simulations of the diffusion of soft spheroidal particles, represented by the Gay-Berne potential, at various volume fractions confirmed the aspect ratio dependence of the diffusion of spheroids. Figure 6B is a plot of the normalised diffusion rate of Gay-Berne prolate spheroids of 1.0, 1.3, 1.6 and 2.0 aspect ratio at volume fractions of up to 55% determined from Brownian (solid symbols) and Langevin (open symbols) dynamics simulations, with fits of Eq. (14) with *κ*, a scaling parameter for *L*(*ϕ*), and *ϕ*_c_ fitted parameters. The extrapolated values of the critical volume fraction *ϕ*_c_ from both simulation methods show an identical dependence on shape (Fig. 6C), starting at a value of 0.565 for *α* = 1.0 (spheres) increasing to a maximum of 0.64 at *α* = 1.64 and then declining.

**Figure 7:**
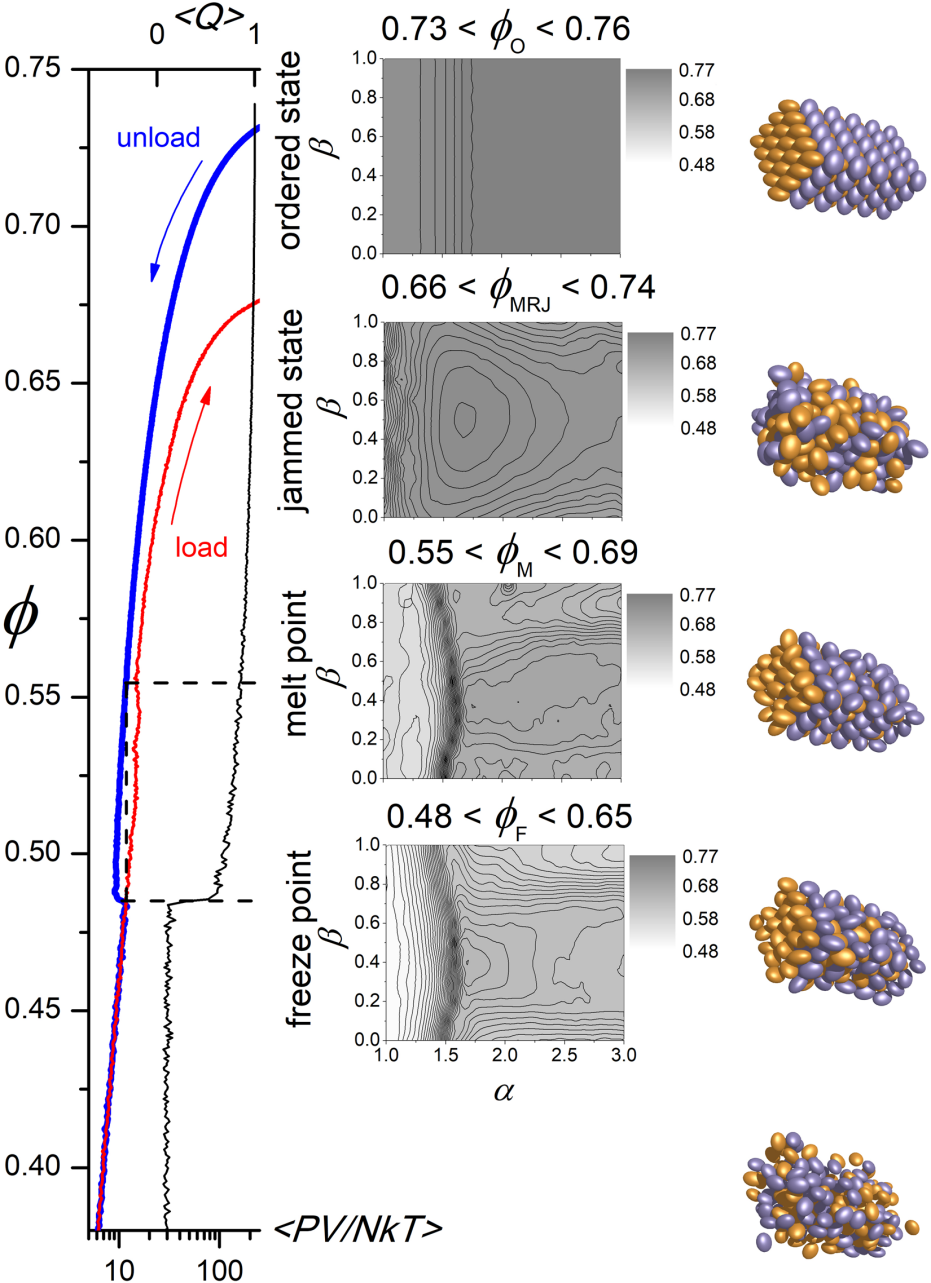
Estimation of the critical freeze *ϕ*_F_, melt *ϕ*_M_, and maximum jammed *ϕ*_MRJ_ volume fractions. The ordered crystal state for each ellipsoid defined by *αβ* (top) is unpacked during which both the pressure of the system (blue) and order (black) are recorded (left). At the freeze point there is a slight increase in pressure as order is lost precipitously. The melt point is taken as the more packed state that exerted the same pressure as the raised value at the freeze point. After unpacking, the system is then packed to determine the maximum random jammed state (left, red).

The crystal unpacking simulations gave values for *ϕ*_F_, *ϕ*_M_ and *ϕ*_MRJ_ for spheres as 0.49, 0.56 and 0.68, respectively (Fig. 7). The value of *ϕ*_G_ ≈ 0.58 lies between *ϕ*_M_ and *ϕ*_MRJ_. *ϕ*_M_ follows a similar trend as *ϕ*_c_ obtained from the Brownian and Langevin dynamics simulations (Fig. 6C), with both starting at 0.56 for *α* = 1.0 and exhibiting a maximum near *α*_*β*=0_ = 1.6. The *ϕ*_MRJ_ for ellipsoids 1 ≤ *α* ≤ 3 and 0 ≤ *β* ≤ 1 has a single maximum value *ϕ*_MRJ_ ≈ 0.73 at *α* = 1.72, *β* = 0.5. The value of *ϕ*_F_ is maximal at 0.64 at *α* = 1.71, *β* = 0.4. The value of *ϕ*_M_ has three maxima in this *αβ* profile, with the highest of *ϕ*_M_ = 0.68 at *α* = 1.72, *β* = 0.36. As an extrapolation, however, we expect a degree of variability in the determination of *ϕ*_M_. Ellipsoids with shape *α* ≈ 1.7, *β* ≈ 0.4 are expected, therefore, to have the highest value of *ϕ*_c_, which suggests that the translational diffusion proteins of a similar shape is least retarded by crowding. Proteins of aspect ratio of *α* ≈ 1.7 and *β* ≈ 0.4 have both optimal diffusion and limited propensity to form a glass when at high concentration. Such heterogenous crowding in the cell can turn diffusion from normal to anomalous, where mean-squared displacement is no-longer linear in time^62^. A more complete model of the dependence of diffusion-limited reactions on cellular concentration that incorporates the subdiffusion due to crowding is required^63,64^, although we expect the dependence on aspect ratio of the diffusing particles to be similar to that found here.

### Proteins are ellipsoidal to maximise their translational diffusion in the crowded cell

**Figure 8:**
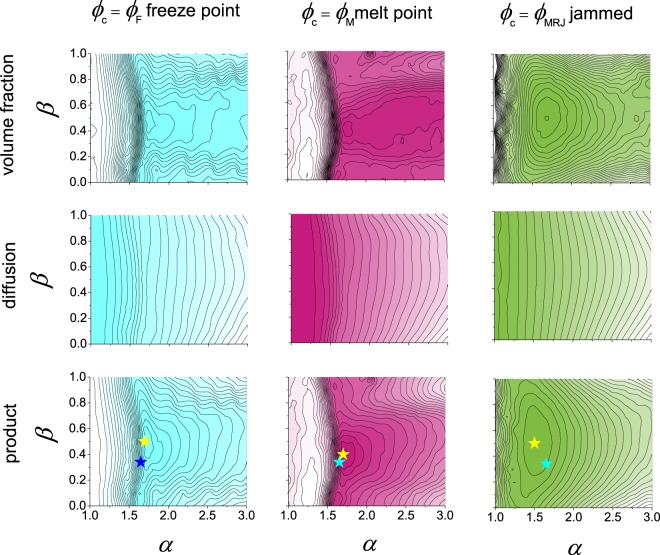
Using the critical volume fraction *ϕ*_c_ as the freeze point *ϕ*_F_ (left, blue), melt point *ϕ*_M_ (middle, red), and jammed point *ϕ*_MRJ_ (right, green) the maximum of the product (bottom row) of the predicted diffusion rate (middle row) and volume fraction (top row) for each ellipsoid *αβ* is found. The maximum of the product is shown as the yellow star and can be compared to the modal value found for the 47677 protein set (blue star).

A biomolecular reaction limited by diffusion has a rate proportional to the product of the relative diffusion constant (i.e. the sum of the diffusion constants of the reactants) and the reactant concentrations. By combining Eqs (11), (12) and (14) we found the product $\varphi {D}_{S}^{L}(\varphi ,{\varphi }_{{\rm{c}}})$ is maximum at *α*_*β*=0_ = 1.58 and *ϕ* = 0.19 for prolate spheroids, and is maximum when *α* = 1.70, *β* = 0.5, *ϕ* = 0.18 using *ϕ*_c_ = *ϕ*_M_, and maximum when *α* = 1.60, *β* = 0.5, *ϕ* = 0.20 for *ϕ*_c_ = *ϕ*_MRJ_ (Fig. 8). Thus, using *ϕ*_c_ of an ellipsoid suspension as either *ϕ*_M_ or *ϕ*_MRJ_ and assuming the diffusive behaviour of proteins at finite concentration remains equal to the ellipsoids of equivalent steric volume, we recover the cell volume fraction of 19% and additionally the optimum protein aspect ratio of *a*/*c* ≈ 1.7 and *b*/*c* ≈ 1.3, similar to that measued of proteins that form dimers (Table 1).

The predicted translational diffusion of the protein at a volume fraction of 19% is 35% of its value at infinite dilution, giving a simple expression for the approximate translational diffusion [×10^−7^ cm^2^ s^−1^] of a protein in the cell at 37 °C, *ρ* = 0.6913 mPa s of

18 $${D}_{{\rm{t}}}^{{\rm{cell}}} \sim \frac{28}{\sqrt[3]{N}}\approx \frac{135}{\sqrt[3]{{M}_{{\rm{p}}}}}.$$

### Ellipsoidal shape helps proteins avoid non-functional interactions

**Figure 9:**
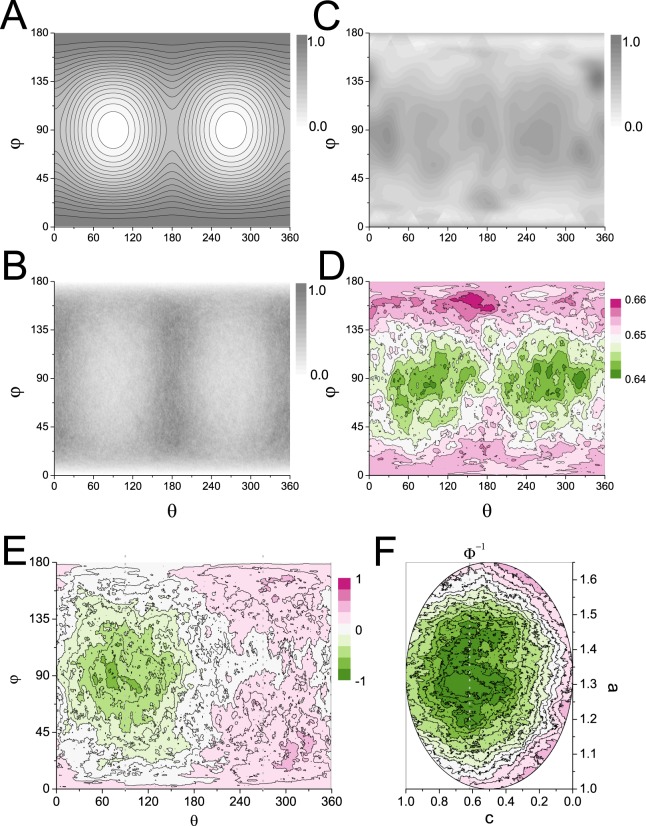
Proteins have surfaces that are nonsticky at the sites where they are most likely to make collisions during diffusion. (**A**) The effective translational diffusion over short time of points on the surface of a tumbling ellipsoid, defined by two angles *θ* from the *a* axis and *φ* from *b*, is lowest (light) at the poles of the *c* axis, the flattest face, and highest (dark) at the poles of the *a* axis. (**B**) Plot of the normalized distribution of collision frequency (low light to high dark) for each *θφ* point on the surface of a diffusing ellipsoid. (**C**) Plot of the frequency distribution of the location of points at the centre of the interface between monomers in a protein dimer (low light to high dark). (**D**) Plot of the average radial extent values (Fig. 3A) for the residues found on the surface of the monomers in protein dimers. (**E**) The most conserved residues on the surface of homologs are coincident with the centre of the pole at *c* where binding and active sites are common. (**F**) The *a*/*c* cross-section through the evolutionary rate data reveals that mutations of the faster-evolving residues on the surface away from the active site facilitate changes in the core.

Figure 9B shows the normalized frequency distribution in *θφ* space of the location of collisions between ellispoids (*α* = 1.78, *β* = 0.44) undergoing Brownian dynamics. Each collision at *θφ* on the surface of the ellipsoid is represented by a Gaussian spot with variance 10° arc. The locations of the collisions are distributed unevenly across the surface with the fewest collisions made at the poles of the *c* axis.

The distribution is similar in form to that of the effective translational diffusion constants over short times (i.e. dominated by the rotation) *D*_t*p*_(*x*, *y*, *z*) of points across the surface of the ellipsoid (Fig. 9A), calculated as

19 $${D}_{{\rm{t}}p}(x,y,z)\approx {D}_{t}+\frac{1}{3}[{D}_{{\rm{r}}a}({y}^{2}+{z}^{2})+{D}_{{\rm{r}}b}({x}^{2}+{z}^{2})+{D}_{{\rm{r}}c}({x}^{2}+{y}^{2}\mathrm{)].}$$

Points that are translating the quickest, i.e. the poles of the *a* axis, make more contacts than those which are translating the slowest, i.e. the poles of the *c* axis. Molecular dynamics simulations support this importance of rotational diffusion in directing collisions between proteins^65^.

We compared the collision probabilities to the points of the centres of protein binding patches measured from the 3D Complex database^16^. We calculated the ellipsoid of equivalent steric volume of each partner in a binary complex and determined their centre of contact by shrinking the ellipsoids until their surfaces just touched. Figure 9C shows the distribution of the *θφ* contact points of 9958 protein pairs, where each point is mapped as a Gaussian-spot with variance 10° arc across the surface. Figure 9D shows the map of the average extent to which the amino acid at the contact point is normally found at the surface (Fig. 3A), which reflects the patch stickiness as calculated by Levy *et al*.^19^. Protein-protein interaction sites were found predominately on the face of the poles of the *c* axis, at residues that are generally normally found more buried in a protein. Whilst the face at *c* presents the greatest surface area it is the area involved in the fewest collisions during diffusion. An ellipsoidal shape to protein directs random collisions between them to nonsticky regions of their surface and thereby limits the formation of non-functional interactions and aggregation.

A protein’s interaction or active site is expected to be the most conserved during evolution, since mutation of a residue at the site is more likely to lead to loss of function than a mutation elsewhere on the surface^19,34^. Therefore, we expected the residues at the poles of the *c* axis to be most conserved in homologs across species of known phylogeny. Following the method of Tóth-Petróczy and Tawfik^34^ we calculated the evolution rates-per-position of 382 protein domains of known structure in orthologs in 10 yeast species. In Fig. 9E we plot the average rates of the surface residues mapped onto *θφ* (the protein is rotated around *a* by 0° or 180° so the face at *c* with the slower rates is at 0 < *φ* < 180). The residues around the centre of the *c* face at *θ* = 90°, *φ* = 90°, coincident with the location of the active site most often, were found to have an evolution rate around half that of the other surface residues. A cross section taken through the data taken at *φ* = 90° highlights the association of evolutionary rates between the residues at the surface and those at the core. The evolutionary rates of residues decrease with their distance from the surface with core residues exchanging on average fourfold slower than those on the surface (away from the centre of *c* at *θ* = 90°, *φ* = 90°). The core region of the protein that is most conserved is offset from the centre of the protein towards the interface. This too is as expected following the surface–core association of evolutionary rates revealed by Tóth-Petróczy and Tawfik^34^. Mutations of the faster evolving residues on the surface away from the active site facilitate changes in the core. The rate of evolution decreases with distance from the surface, converging on a point that is offset the *c* axis (coincident with Φ). If core mutations were facilitating surface changes we would expect the most conserved residues to be found at the centre of the protein, which is not the case.

## Summary

We have shown that proteins are generally aspherical with an aspect ratio distributed around 1.6; a value close to the Golden ratio. We have shown than proteins of random sequence greater than 270 residues in length are likely to be ellipsoidal irrespective of secondary structure and that a simple model of a protein of 400 residues has a shape similar to that most common in the PDB.

We have shown that the translational and rotational diffusion of proteins can be accurately modelled by considering the protein as a triaxial ellipsoid of equivalent steric volume. We have shown that the translational diffusion of such ellipsoids decreases with increasing concentration and that the critical volume fraction corresponding to the glass transition, where translational diffusion becomes negligible, is dependent on the shape of the ellipsoid. We found that the shape of ellipsoid that gives the highest predicted value for the critical volume fraction is coincident with the modal value found for proteins. We suggested, therefore, that proteins have a shape that maximizes their translational diffusion within the cell and limits the likelihood of gelation at high concentration.

We studied the location of contacts between diffusing ellipsoids and found a correlation between the location where collisions are least likely to occur and where the protein/protein interface is found in protein dimers. We found the same association between the type of residues found at these interaction sites as others^19^, and showed in a series of proteins that the residues that are found at these sites have evolved less quickly than those on other regions of the protein where random collisional contacts are more likely to occur. We suggested that proteins have evolved a shape which directs non-functional random collisions away from their sites of interaction to non-sticky residues that are least conserved.
